# Nitrogen application in pod zone improves yield and quality of two peanut cultivars by modulating nitrogen accumulation and metabolism

**DOI:** 10.1186/s12870-024-04725-1

**Published:** 2024-01-13

**Authors:** Guanghui Li, Xin Guo, Wei Sun, Lei Hou, Guanghao Wang, Ruizheng Tian, Xingjun Wang, Chunjuan Qu, Chuanzhi Zhao

**Affiliations:** 1 Institute of Crop Germplasm Resources (Institute of Biotechnology), Shandong Provincial Key Laboratory of Crop Genetic Improvement, Ecology and Physiology, Shandong Academy of Agricultural Sciences, Jinan, 250100 China; 2 Linyi Academy of Agricultural Sciences, Linyi, 276012 China; 3 Shandong Peanut Research Institute, Qingdao, 266100 China

**Keywords:** Peanut, Pod zone nitrogen application, Yield, Enzymatic activity, Gene expression

## Abstract

Cultivated peanut (*Arachis hypogaea* L.) represents one of the most important oil and cash crops world-widely. Unlike many other legumes, peanuts absorb nitrogen through their underground pods. Despite this unique feature, the relationship between yield and nitrogen uptake within the pod zone remains poorly understood. In our pot experiment, we divided the underground peanut part into two zones—pod and root—and investigated the physiological and agronomic traits of two peanut cultivars, SH11 (large seeds, LS) and HY23 (small seeds, SS), at 10 (S1), 20 (S2), and 30 (S3) days after gynophores penetrated the soil, with nitrogen application in the pod zone. Results indicated that nitrogen application increased pod yield, kernel protein content, and nitrogen accumulation in plants. For both LS and SS peanut cultivars, optimal nitrogen content was 60 kg·hm^− 2^, leading to maximum yield. LS cultivar exhibited higher yield and nitrogen accumulation increases than SS cultivar. Nitrogen application up-regulated the expression of nitrogen metabolism-related genes in the pod, including nitrate reductase (*NR*), nitrite reductase (*NIR*), glutamine synthetase (*GS*), glutamate synthase (*NADH-GOGAT*), ATP binding cassette (*ABC*), and nitrate transporter (*NRT2*). Additionally, nitrogen application increased enzyme activity in the pod, including *NR*, *GS*, and *GOGAT*, consistent with gene expression levels. These nitrogen metabolism traits exhibited higher up-regulations in the large-seeded cultivar than in the small-seeded one and showed a significant correlation with yield in the large-seeded cultivar at S2 and S3. Our findings offer a scientific basis for the judicious application and efficient utilization of nitrogen fertilization in peanuts, laying the groundwork for further elucidating the molecular mechanisms of peanut nitrogen utilization.

## Introduction

Peanuts are extensively cultivated in over 100 countries and are cherished by consumers worldwide [1]. In 2020, global peanut cultivation covered 32 million hectares, yielding approximately 54 million metric tons, with China contributing more than 18 million metric tons [2].Recognized as a vital cash and oil crop, peanuts contain a substantial oil content in their kernels (40-60%). Moreover, they serve as a staple in the food industry, utilized for butter, soup thickening, nuts, chocolate beans, and sprouts, owing to their rich protein content (20-40%), carbohydrates (10-20%), and bioactive compounds such as flavonoids, vitamins, resveratrol, and anthocyanins [3–5].

Nitrogen (N) stands out as a crucial nutrient and a limiting factor in agricultural systems worldwide [6]. N fertilizer application is a common practice to sustain crop productivity, supporting the rapid global population growth [7], however, excessive use can reduce N utilization efficiency, leading to environmental issues like soil degradation and water contamination [8].

As a legume crop, peanuts acquire nitrogen from both soil through their roots and atmospheric nitrogen fixation via nodules. Nitrogen fixation in peanuts can contribute over 60% of the total nitrogen requirement in poor soil fertility conditions and around 40% in high-yield cultivation [9, 10]. Adequate nitrogen fertilizer application becomes essential for optimizing yield [11].

Typically, the nitrogen fertilizer management strategy for peanuts involves applying all nitrogen fertilizer as basal fertilizer at planting, providing plant nutrient requirements before nodule formation. However, this approach can lead to excessive vegetative growth in early phases due to nitrogen excess and premature senility in reproductive phases due to nitrogen deficiency [12]. In addition, early-stage excess nitrogen inhibits root nodule formation and rhizobium activities, limiting potential symbiotic nitrogen fixation [13]. Excessive nitrogen fertilizer application reduces nitrogen-fixation traits by approximately 50% in each peanut growing season, with more significant effects on J11 and Gangapuri (Spanish types) than NC17 and Robut33-1 (Virginia types) [14]. Virginia cultivars outperform Spanish types in nodulation, nitrogen fixation, specific activity, growth, pod yield, and harvest index. Nitrogen assimilation and nitrogen use efficiency (NUE) in peanuts increase within a certain nitrogen application range but decrease with excess nitrogen fertilizer application [15]. This highlights the necessity for efficient nitrogen transport and assimilation to drive growth and development when enhancing nitrogen acquisition [16]. There are notable differences in nitrogen uptake, transport, and assimilation efficiency among different peanut cultivars [17]. Previous researches mainly focused on root uptake and nodule fixation, however, nitrogen fertilizer assimilation and application by pods have been scarcely explored.

Peanuts are geocarpic crops, exhibiting the unique trait of pushing the ovary into the soil for pod development after fertilization [18]. During pod development, both gynophores and underground pods participate in nutrient uptake. At the pod filling stage, over 90% of Ca and 40% of Zn are directly absorbed by pods from the soil [19, 20], highlighting the crucial role of sufficient calcium in the pod zone for pod development, yield formation, and nutritional quality [21]. The late podding stage represents a critical period for nitrogen (N) accumulation, where the capacity of root N uptake and nodule N fixation significantly decreases, limiting pod production [22]. Previous studies have demonstrated that a substantial portion of N fertilizer applied to the pod zone can be absorbed by the pod, with a notable proportion remaining in the fruit parts [23]. Our earlier investigation into peanut pod N uptake, involving soil separation between roots and pods, revealed that the application of 60 kg/hm² of N in the pod zone resulted in the highest pod yield for FH1 (large-seeded type) [24].

Effective management and appropriate use of nitrogen fertilizers in the pod zone present a promising strategy to boost peanut productivity and optimize nitrogen utilization efficiency.Peanut cultivars are commonly categorized into large-seeded and small-seeded based on seed size/weight. Large-seeded cultivars generally have a more extended pod filling period, leading to increased nitrogen uptake and accumulation compared to small-seeded ones [25]. However, information regarding the responses of pod yield in cultivars with diverse seed sizes to pod zone N fertilizer application is currently lacking, necessitating further investigation. Our study aims to explore the responses of pod yield and nitrogen accumulation in two peanut cultivars with distinct pod sizes to pod zone N fertilizer application. Additionally, we seek to unravel the physiological mechanisms underlying nitrogen absorption. The outcomes of this research will furnish valuable insights for advancing nitrogen precision management in peanut cultivation.

## Materials and methods

### Experimental design and treatments

The pot experiments were carried out at the Field Crop Research Station of Shandong Academy of Agriculture Sciences, Jinan, China. The soil type was a silty loam, with organic matter 13.7 g kg^− 1^, alkali-hydrolyzable nitrogen 80.2 mg kg^− 1^, available phosphorus 42.2 mg kg^− 1^, available kalium 238.5 mg kg^− 1^, exchangeable calcium 5.2 g kg^− 1^, in 0 ~ 20 cm tillage layer, and with pH 6.5.

Cylindrical pots with the height of 35 cm and diameter of 40 cm were used. Each pot was filled with air-dried soil from the 0 ~ 20 cm tillage layer, resulting in a soil weight of 25 kg per pot.Nitrogen fertilizer as urea at the rate of 1.51 g N pot^− 1^, phosphorus fertilizer in the form of triple superphosphate at the rate of 1.13 g P_2_O_5_ pot^− 1^ and potassium fertilizer in the form of potassium sulphateat 1.88 g K_2_O pot^− 1^ were applied. Then water was supplied to saturation for each pot before planting.

**Fig. 1:**
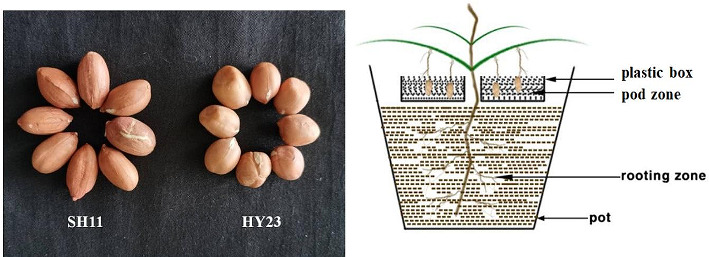
The seeds of two peanut cultivars and schematic diagram of the separation of the rooting zone and the pod zone

Two peanut cultivars, SH11 (LS) and HY23 (SS), were used in this study (Fig. 1). Three plump seeds were sown in each pot, and two uniform seedlings were retained per pot at seven days after germination. Before flowering, designed plastic boxes were placed into the pots, filled with 4 kg of clean river sand, serving to separate the pod zone from the rooting zone (Fig. 1). This setup allowed for the growth of peanut pods and roots in different nutritional environments. The experiment utilized a split-plot design, with peanut cultivars as the main plots and nitrogen treatments in the pod zone as the sub-plots. Before the gynophore penetrated into the soil, nitrogen fertilizer (urea) was applied in the pod zone at rates of 0 g (control), 0.38 g, 0.75 g, and 1.13 g N per pot, corresponding to 0, 30, 60, and 90 kg·hm-2, respectively. Nitrogen-free nutrient solution was applied to the pod zone along with fertilization, following the methods described by Inanaga et al. [23]. Each treatment consisted of ten replicate pots. The trials were sown on May 15th, 2020, and the plants were harvested on September 20th, 2020.

For gene expression analysis and enzyme activity study, pod samples were collected at 10, 20, and 30 days after gynophores penetrated into the soil (S1, S2, and S3), frozen with liquid nitrogen, and stored at -80 °C. Plant samples were taken at 20 days after gynophores penetrated soil (20DAP), 40 DAP, and 60 DAP, representing three key stages of pod development: pod expanding, rapid filling, and harvest stages, respectively. The plants were divided into root, stem, leaf, shell, seed, and gynophore. All samples were washed, fixed, dried at 105 °C for 0.5 h, and then dried at 80 °C to constant weight in a drying oven. Dry weight and total nitrogen in each part were determined.

### Determination of nitrogen content

The dried samples were finely ground using a plant grinder, and the nitrogen content was subsequently assessed using the Kjeltec 2300 automatic nitrogen determination instrument.

### Determination of protein and total amino acids content

The protein and total amino acid contents were analyzed using near-infrared spectroscopy (DA7250, Perten, Stockholm, Sweden). The instrument, equipped with data acquisition and analysis software, allows for the creation of standard curve models to analyze four quality indexes, including crude fat, protein, fatty acids, and amino acids. Plump and intact peanut kernels were placed in a spinning ring cup with a 7.5 diameter, ensuring a flat surface. To mitigate the impact of kernel size, each sample underwent two scans, involving emptying and refilling the ring cup before the second scan.

### Determination of NR enzyme activity

NR activity was assessed following the method of Khator and Shekhawat [26], with minor adjustments. Fresh samples (0.5 g) were crushed using a pre-cooled mortar in 4 mL of 50 mM potassium phosphate buffer (pH 8.0), which contained 25 mM cysteine, 1 mM EDTA, and 3% (w/v) BSA at 0 ~ 4℃. The homogenate was then centrifuged at 12,000×g for 20 min at 4 °C, and the resulting supernatant was utilized for NR activity analysis. The assay mixture, composed of 0.4 mL enzyme solution, 1.2 mL potassium phosphate buffer (0.1 M), 0.2 mL potassium nitrate (50 mM), and 0.2 mL NADH (3 mM), was incubated for 30 min at 25 °C. Following incubation, 1 mL sulphanilamide (58 mM) and 1 mL α-naphthylamine (14 mM) were added to the reaction mixture and incubated for an additional 15 min. The solution was then centrifuged at 4000×g for 5 min, and the supernatant was collected for absorbance measurement at 540 nm. A blank assay mixture without NADH was included for reference.

### Determination of GS and GOGAT enzyme activities

Fresh samples (0.5 g) were homogenized in a pre-chilled mortar with 5 mL Tris–HCl buffer (pH 8.0) containing 50 mM Tris-HCl, 10 mM MgSO_4_, 10 mM EDTA, and 10 mM cysteine. Extracts were centrifuged at 12,000×g for 15 min at 4 ℃, and the resulting supernatants were utilized for the assay of GS and GOGAT.

GS activity was determined following the method of O’Neal and Joy [27]. The reaction mixture consisted of 1 ml reaction system (50 mM Tris-HCl, 20 mM MgSO_4_, 5 mM NH_2_OH, 8 mM ATP, 1 mM EDTA, 80 mM α-glutamate) and 0.5 ml enzyme solution. The mixture was incubated at 35 ºC for 30 min and terminated by adding 0.5 mL FeCl_3_. After centrifugation at 5000×g for 15 min, the absorbance was recorded at 540 nm using a UV-spectrophotometer. GS activity was calculated based on γ-glutamyl hydroxamate, with one unit of enzyme activity defined as the production of 1 µmol γ-glutamyl hydroxamic acid per mg of fresh tissue per hour per mL of the reaction system.

For GOGAT activity, 0.3 mL enzyme solution was added to 1.2 mL reaction solution consisting of 0.1 M potassium phosphate (0.1 mL), 2 mM α-oxoglutarate (0.5 mL), 0.2 mM NADH (0.2 mL), and 10 mM L-glutamine (0.4 mL). The mixture was incubated at 30 ºC for 30 min and terminated by a boiling water bath for 30 s. The absorbance was immediately read at 340 nm. The reaction solution without L-glutamine served as a control, with one unit of GOGAT activity defined as the decrease of 1 µmol NADH per min.

### Expression analysis of genes for nitrogen uptake and metabolism in pod

Total RNA extraction was conducted using the Trizol Reagent kit (TaKaRa, Inc., Dalian, China) following the manufacturer’s instructions. Three biological replications were employed, and RNA samples underwent DNase I treatment to eliminate genomic DNA contamination. Agilent 2100 and NanoDrop were utilized to assess RNA quality and purity. Total RNA from each sample was employed for mRNA enrichment with Oligo (Dt) and subsequent cleavage into short fragments (~ 200 nt) in a fragmentation buffer. Reverse transcription, using a random hexamer primer to obtain the first-strand cDNA, and synthesis of the second strand of cDNA using buffer, dNTPs, RNaseH, and DNA polymerase were performed. After end repair and the addition of sequencing adaptors, the cDNA fragments underwent PCR amplification with gene-specific primers. The gene-specific primers, designed using Perl Primer software, are listed in Table 1. qRT-PCR was executed using the SYBR Green Pro Taq HS premixed qPCR kit in the 7500 Real-Time PCR System. The thermal cycle parameters were 94 °C for 10 min, followed by 40 cycles of 94 °C for 15 s and 60 °C for 1 min in a 20 mL volume. Three biological replications were conducted for each reaction, with the peanut actin gene serving as an internal reference. The relative gene expression level was calculated using the 2^−ΔΔCT^ method.

**Table 1:** Real-time PCR primers information

Gene name	Primer Sequence(5’-3’)
NR	F: GAAACGCATCATAGTTACACC
	R: GATCAAATACTCTGGCTTGTACC
NiR	F: CGGAAATCTTGAAAGGTCTG
	R: TATCAACAATCTCACGAGGG
NRT2	F: TAACTACACCCTCCTTCAATTCTC
	R: GTTGGTTTCTTGCTTGTGTC
GS	F: TTCTGTTGGTATCTCTGCTG
	R: TGTGCTGTAGTTAGTGTGAG
NADH-GOGA	F: ATAACACAACCTTCCTTTCCAC
	R: TCAGATAACTTCTCACCACCC
ABC	F: TCTACTTCTGCTTCACTTCGG
	R: CATCTTTGCTATTTGCCTTCGT

### Statistical analysis

All parameters were measured in a minimum of three replications and are presented as means ± standard deviation. The average for each trait was computed using Microsoft Excel 2010 and visualized with Sigmaplot 10.0. Duncan’s multiple range test, facilitated by SPSS Statistics 23, was employed to identify significant differences between treatments (*P* < 0.05). Correlation analysis was conducted using OmicShare tools for data analysis.

## Results

### Application of nitrogen in pod zone increased dry matter accumulation and yield

To investigate the impact of nitrogen on peanut yield, a specialized container was devised to segregate the peanut’s underground portions into two zones: the pod zone and the root zone (Fig. 1). Varied concentrations of nitrogen fertilizer were applied to the pod zone of two peanut cultivars. Our findings revealed that nitrogen application enhanced dry matter accumulation throughout the pod development stage for both peanut cultivars, with a more pronounced positive effect on SH11 compared to HY23 (Table 2). In comparison to the control, dry matter accumulation per plant increased by 5.93–9.44% for SH11 and 4.22–7.65% for HY23 at 60 DAP. Notably, the application of 90 kg·hm^− 2^ and 60 kg·hm^− 2^ resulted in the maximum dry matter accumulation for SH11 and HY23, respectively. Both cultivars exhibited a significant increase in pod yield under pod zone nitrogen application compared to the control (Table 2). The pod yield of SH11 and HY23 increased by 8.11–11.30% and 4.68–8.76%, respectively, in comparison to the control. The 60 kg·hm^− 2^ treatment yielded the maximum pod yield for both SH11 and HY23 (Table 2).

**Table 2:** Dry matter accumulation and pod yield per plant under different nitrogen applicationin pod zone

Cultivars	Pod zone nitrogen application (kg·hm^− 2^)	Dry matter accumulation per plant(g)			Yield per plant (g)
		20 DAP	40 DAP	60 DAP	
SH11	0	34.62 ± 2.37a	43.54 ± 0.99b	48.82 ± 0.68c	22.60 ± 0.64b
	30	37.00 ± 1.84a	45.68 ± 1.77ab	51.72 ± 0.64b	24.43 ± 0.78a
	60	37.62 ± 1.52a	46.65 ± 0.56a	53.38 ± 0.27a	25.15 ± 0.25a
	90	36.25 ± 0.48a	45.91 ± 0.96a	53.43 ± 1.13a	24.53 ± 0.59a
HY23	0	30.78 ± 0.26b	40.50 ± 0.67a	40.24 ± 0.99b	19.74 ± 0.60b
	30	31.76 ± 0.70b	40.94 ± 0.62a	41.94 ± 0.71a	20.67 ± 0.46ab
	60	33.76 ± 1.29a	41.85 ± 0.71a	43.32 ± 0.68a	21.47 ± 0.61a
	90	33.15 ± 1.07a	41.96 ± 0.96a	42.90 ± 0.79a	20.81 ± 0.37ab

*Note*: Data are means ± SD of three replicates each containing five plants independently. Different lowercase letters in the same column indicate significantly different between different nitrogen application ratesin the same cultivarat *p* < 0.05 according to Duncan’s multiple range test. DAP means the days after peanut gynophores penetration soil

### Application of nitrogen in pod zone affected the nitrogen contentof whole plants

We observed a significant rise in nitrogen content for entire plants with the application of nitrogen in the pod zone (Table 3). Nitrogen accumulation consistently increased during the pod filling process in SH11, while remaining relatively stable in HY23, compared to the control. The surge in nitrogen accumulation for HY23 (7.29%~16.58%) was slightly higher than that for SH11 (8.79%~12.14%) at 20 DAP. Notably, a more substantial increase in nitrogen accumulation was noted in SH11 compared to HY23 at 60 DAP, with an increase of 13.30%~27.83% in SH11, while HY23 experienced a rise of 8.05%~15.64%. The maximum increases in nitrogen accumulation for SH11 and HY23 were observed with treatments of 90 kg·hm^− 2^ and 60 kg·hm^− 2^, respectively.

**Table 3:** Nitrogen accumulation per plant under different nitrogen application in pod zone

Cultivars	Pod zone nitrogen application (kg·hm^− 2^)	Nitrogen accumulation per plant (mg)			Increase over CK (%)		
		20 DAP	40 DAP	60 DAP	20 DAP	40 DAP	60 DAP
SH11	0	690.6 ± 32.6b	950.4 ± 27.7c	1017.4 ± 40.8c	-	-	-
	30	751.3 ± 41.4ab	1043.6 ± 41.8b	1152.7 ± 15.8b	8.79 ± 0.84a	9.81 ± 2.56b	13.30 ± 4.23b
	60	774.1 ± 45.7a	1148.3 ± 21.2a	1282.8 ± 10.0a	12.09 ± 2.22a	20.83 ± 3.75a	26.08 ± 4.09a
	90	774.4 ± 10.3a	1141.6 ± 34.1a	1300.6 ± 43.4a	12.14 ± 4.46a	20.13 ± 3.79a	27.83 ± 2.27a
HY23	0	577.3 ± 20.3b	761.6 ± 25.9c	832.6 ± 16.1c	-	-	-
	30	619.4 ± 29.2ab	820.6 ± 22.0b	899.6 ± 15.3b	7.29 ± 2.62b	7.75 ± 3.08b	8.05 ± 0.64b
	60	669.3 ± 38.1a	871.2 ± 8.2a	962.8 ± 43.2a	15.95 ± 2.70a	14.40 ± 3.16ab	15.64 ± 4.16a
	90	673.0 ± 30.8a	894.3 ± 10.22a	952.6 ± 38.6a	16.58 ± 1.73a	17.43 ± 3.97a	14.41 ± 2.45a

*Note*: Data are means ± SD of three replicates.Different lowercase letters in the same column indicate significantly different between different nitrogen application ratesin the same cultivar at *p* < 0.05 according to Duncan’s multiple range test. DAP means the days after peanut gynophores penetration soil

### Application of nitrogen in pod zone increased the protein content of seeds

**Fig. 2:**
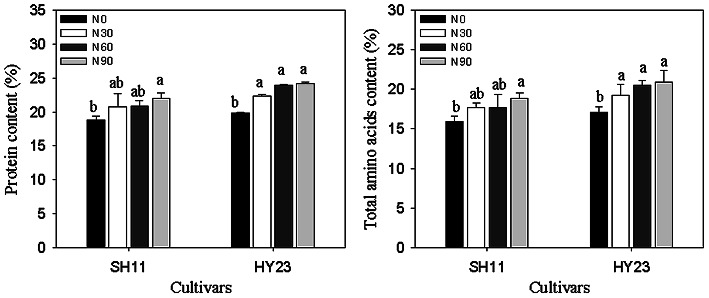
Contents of protein and total amino acids of peanut kernels under different nitrogen applicationin pod zone in SH11 and HY23. Values are shown as means ± SD of three independent biological sample. Different lowercase letters in the bar graph indicate significantly different between different nitrogen application ratesin the same cultivar at *P* < 0.05 according to Duncan’s multiple range test. N0, N30, N60, N90 meaning that nitrogen application ratesin pod zone were 0, 30, 60 and 90 kg·hm^− 2^, respectively

Protein and total amino acid content are crucial quality indicators for peanuts. Our observations revealed a consistent increase in protein and total amino acid content in the kernels of both cultivars with the application of nitrogen in the pod zone, ranging from 0 to 90 kg·hm^− 2^ (Fig. 2). In comparison to the control, SH11 exhibited an increase of 10.43%~17.56% in protein content and 12.41%~21.51% in total amino acids content. The patterns of increase in these two nutritional components in HY23 were essentially parallel to those in SH11.

### Application of nitrogen in pod zone affected the activities of NR, GS, GOGAT

**Fig. 3:**
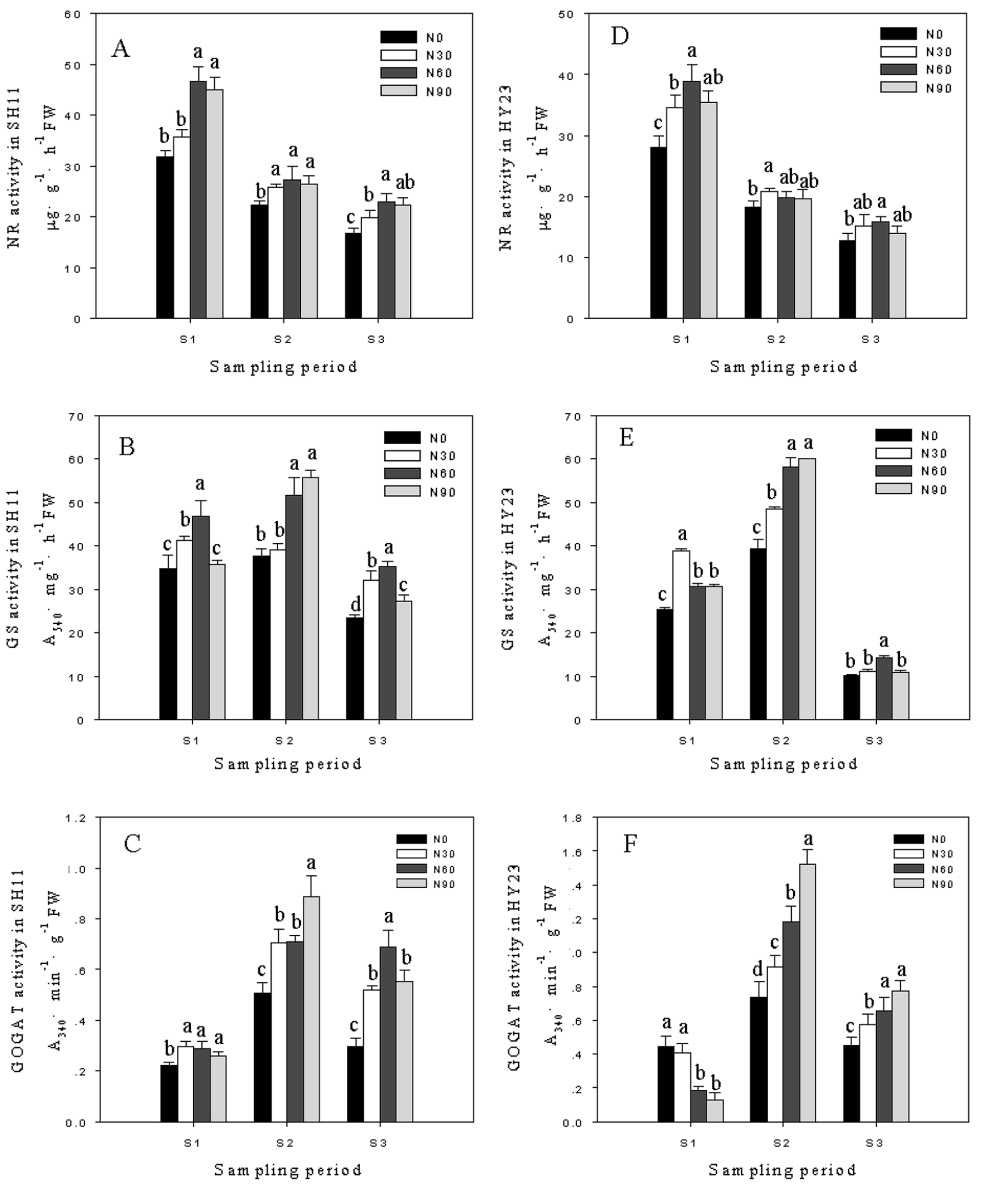
The pod enzymatic activities of NR, GS, GOGAT under different nitrogen applicationin pod zone in SH11(**A**, **B**, **C**) and HY23 (**D**, **E**, **F**). Values are shown as means ± SD of three independent biological sample. Different lowercase letters in the bar graph indicate significantly different between different nitrogen application ratesin the same cultivar at *P* < 0.05 according to Duncan’s multiple range test. N0, N30, N60, N90 meaning that nitrogen application ratesin pod zone were 0, 30, 60 and 90 kg·hm^− 2^ respectively. S1, S2, S3 represent 10, 20, 30 days after peanut gynophores penetration soil

With pod development, NR activity exhibited a declining trend in both the control and nitrogen-applied treatments (Fig. 3-A, D). In comparison to the control, pod zone nitrogen application led to a significant increase in NR activity at all three stages, with higher levels observed in SH11 than in HY23. At S1, NR activities increased by 12.34%~46.99% in SH11 and 23.20%~38.83% in HY23; at S2, the increases were 16.34%~22.24% in SH11 and 8.28%~14.52% in HY23; at S3, the increases were 18.54%~36.40% in SH11 and 8.64%~24.23% in HY23, with the maximum increases observed at the 60 kg·hm^-2^ treatment in both cultivars (Fig. 3-A, D).

For GS activity, it was significant induced by application of nitrogen in pod zone for both cultivars (Fig. 3-B, E). At S1, the highest increase in GS activity was observed in SH11 (35.06%) and HY23 (52.38%) under the 60 kg·hm^− 2^ and 30 kg·hm^− 2^ treatments. At S2, GS activities in both cultivars gradually increased with the rising application of nitrogen in the pod zone. At S3, GS activities in the 60 kg·hm^− 2^ treatment were significantly higher than in others for both cultivars, increasing by 49.94% in SH11 and 38.64% in HY23 compared to the control.

The changing patterns of GOGAT activity were similar to that of the GS activity (except for the values at S1 in HY23) (Fig. 3-C, F). Compared to the control, the maximum increases in GOGAT activity in SH11 (76.02%~133.27%) were observed at S3, while in HY23 (24.62%~106.59%), they occurred at S2.

### Relative gene expression levels of *NR*,*NIR*,*ABC*,*NRT2*,*GS* and *NADH-GOGAT*

**Fig. 4:**
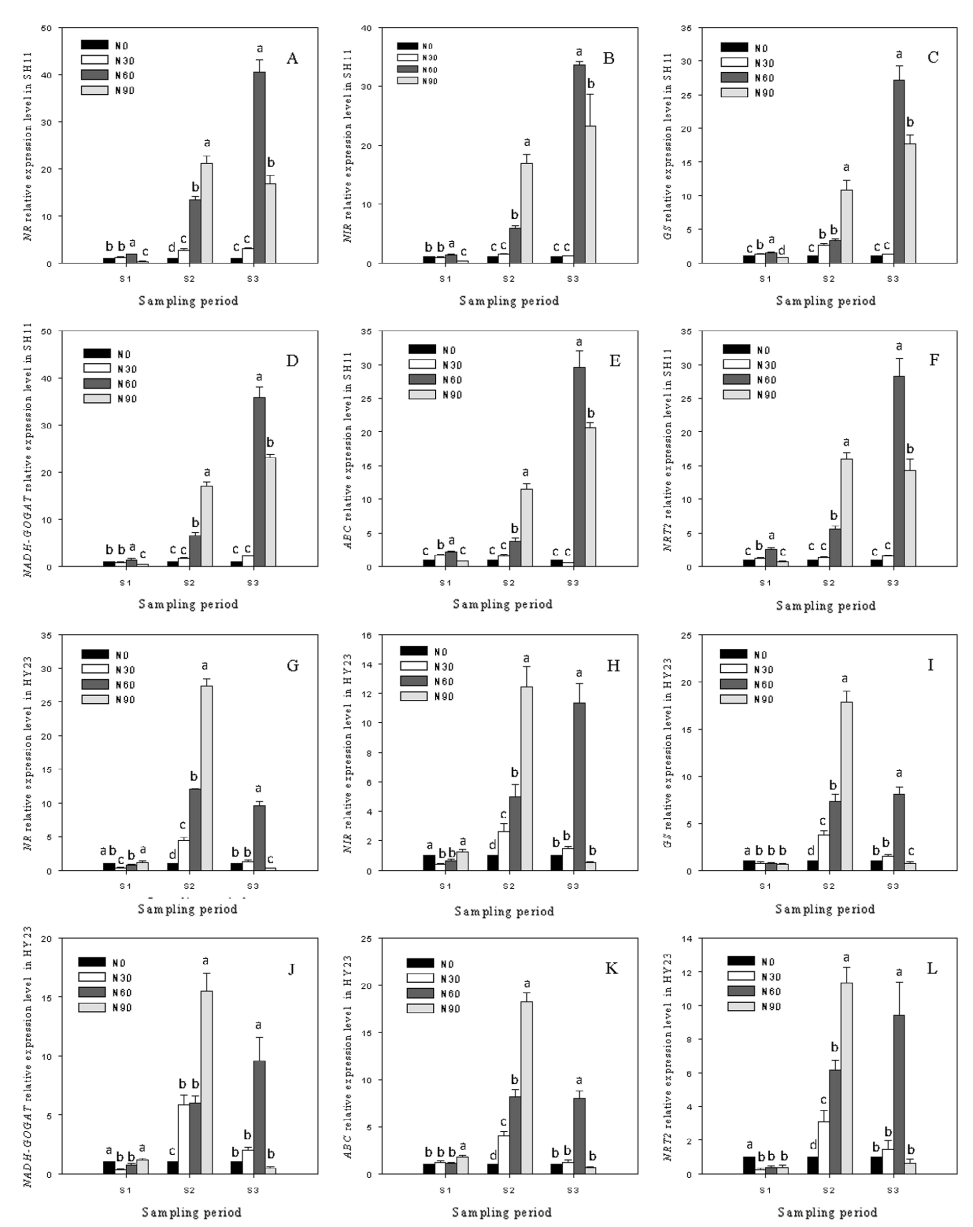
Relative genes expressions of *NR*, *NIR*, *GS*, *NADH-GOGAT*, *ABC* and *NRT2* under different nitrogen applicationin pod zone in SH11(**A**, **B**, **C**, **D**, **E**, **F**) and HY23 (**G**, **H**, **I**, **J**, **K**, **L**). Values are shown as means ± SD of three independent biological sample. Different lowercase letters in the bar graph indicate significantly different between different nitrogen application ratesin the same cultivar at *P* < 0.05 according to Duncan’s multiple range test. N0, N30, N60, N90 meaning that nitrogen application ratesin pod zone were 0, 30, 60 and 90 kg·hm^− 2^ respectively. S1, S2, S3 represent 10, 20, 30 days after peanut gynophores penetration soil

The qRT-PCR method was employed to assess the expression of genes related to nitrogen metabolism. In general, the application of nitrogen in the pod zone induced the expression of genes involved in nitrogen metabolism, particularly at S2 and S3 (Fig. 4). The relative expression levels of *NR*, *NIR*, *ABC*, *NRT2*, *GS*, and *NADH-GOGAT* in SH11 were up-regulated at S1 under pod zone nitrogen application compared to the control. However, they exhibited slight down-regulation at the 90 kg·hm^− 2^ treatment. Similar results were observed in HY23 at the same stage. The increasing trends in the relative expressions of the six genes were consistently observed with the rising nitrogen application at S2, showing up-regulation of more than 10 folds in both cultivars compared to the control at the 90 kg·hm^− 2^ treatment. By S3, the expression of the six genes continued to be up-regulated in SH11. However, in HY23, there was an extreme decrease compared to those at S2, especially without the 60 kg·hm^− 2^ treatment. At S3, the maximum up-regulations of the relative expressions of the six genes were observed with the 60 kg·hm^− 2^ pod zone nitrogen application in both cultivars, all up-regulated 27 folds in SH11 and 7.99 ~ 11.35 folds in HY23. The 90 kg·hm^− 2^ treatment up-regulated gene expression by 14.26 ~ 23.30 folds in SH11; however, their expression level decreased in HY23 compared to the control.

### Correlation analysis

**Fig. 5:**
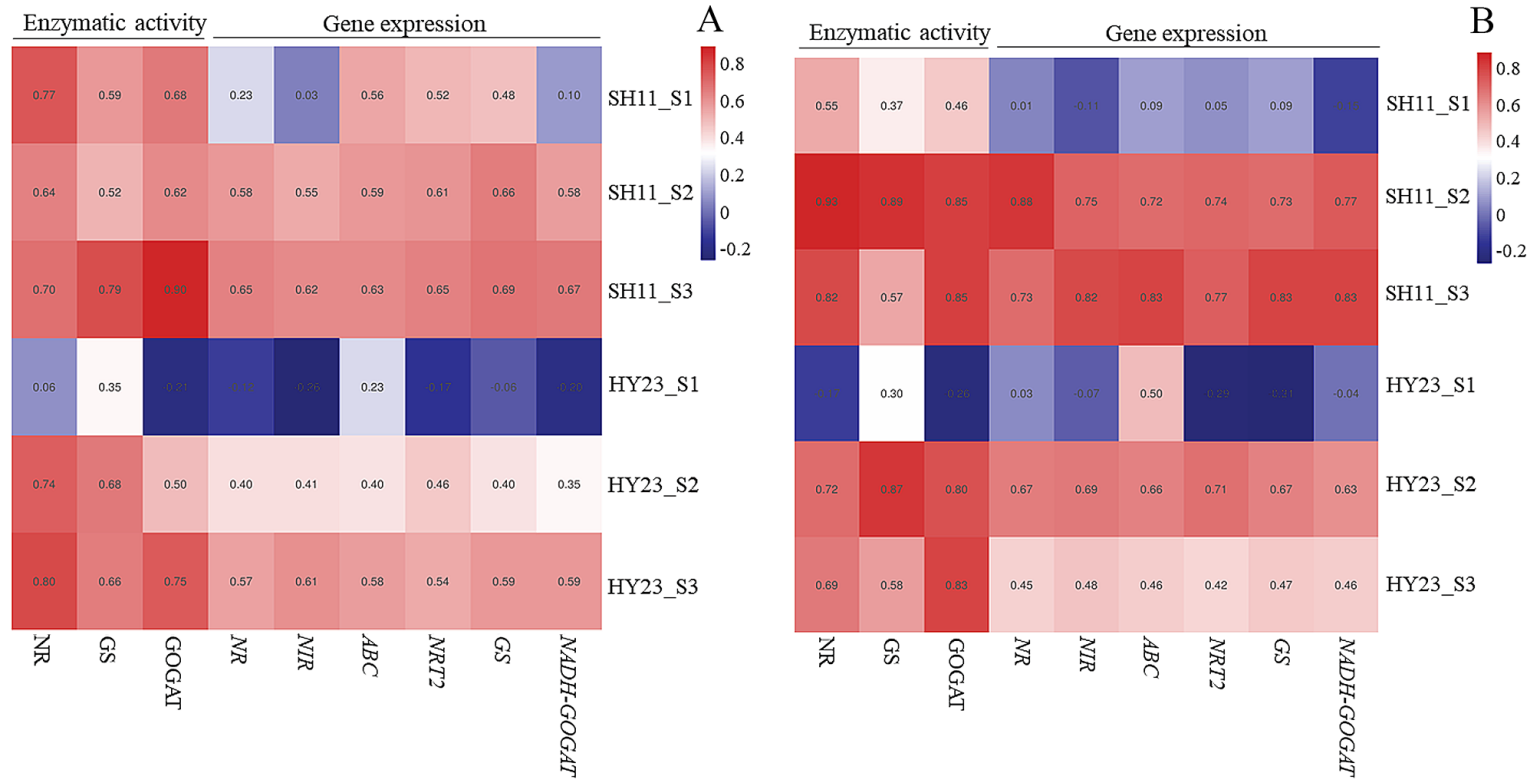
The Heatmap of correlation analysis between key genes and enzyme activities related to nitrogen metabolism in peanut pod and yield per plant (**A**), and nitrogen accumulation per plant (**B**). SH11 and HY23 were two peanut cultivars. S1, S2, S3 represent 10, 20, 30 days after peanut gynophores penetration soil

To further assess the impact of nitrogen application in the pod zone on yield, we conducted correlation analysis between pod yield and nitrogen metabolism traits, encompassing the enzyme activities of *NR*, *GS*, *GOGAT*, and gene expression (Fig. 5-A). A significant positive correlation (*P* < 0.05) was identified between nitrogen metabolism traits and pod yield in SH11 at S2 and S3, and in HY23 at S3 only. The correlation coefficients between nitrogen metabolism traits and pod yield were highest at S3 for both cultivars. However, there was no significant correlation between nitrogen metabolism traits and plant nitrogen accumulation under pod zone nitrogen application conditions in the two cultivars at S1 (Fig. 5-B). Remarkably, nitrogen metabolism traits exhibited a robust positive correlation (*P* < 0.01) with plant nitrogen accumulation in SH11 at S2 and S3, while this correlation was observed in HY23 at S2 only. Pod zone nitrogen applications appeared to stimulate nitrogen uptake and transformation in pods, potentially contributing to the elevated plant nitrogen accumulation and pod yield.

## Discussion

As the fourth-largest edible oil and the third most important vegetable protein globally [28], peanuts are nutrient-intensive crops. The supply of nitrogen (N) fertilizer and its balance with plant development requirements are key factors in peanut production [29]. The accumulation of dry matter and pod yield in peanuts is closely linked to total N accumulation, displaying a quadratic relationship with N application rates under N deficiency conditions [30]. However, excessive N application can significantly reduce yields and dry matter [31]. Achieving a 50% N split ratio at flowering-pegging or pod-filling stages has been shown to significantly enhance peanut production and pod yield, ensuring sufficient N for reproductive growth [22, 32]. Despite symbiotic N fixation, there is still insufficient N to meet the demands of plant growth and development after flowering. Previous research has indicated that pod zone N application increases peanut yield, particularly addressing root zone N deficiency [24]. Our study further confirms that applying N in the pod zone significantly increases pod yield, with the 60 kg·hm^− 2^ treatment achieving maximum yield. Excessive N application might lead to an imbalance in the C/N ratio, promoting vegetative development [33]. Larger-seeded cultivar SH11 exhibited higher pod yield and a more substantial increase than the smaller-seeded cultivar HY23 under the same pod zone N application conditions. This suggests that larger-seeded peanut cultivars are more sensitive to N, maintaining higher yields and N accumulation than smaller-seeded varieties [14, 25]. Our findings align with those in rice, where N application had a more pronounced effect on high-yield hybrid rice cultivars than on straight cultivars [34].

Peanut shells can absorb mineral elements from the soil and translocate them to vegetative tissues [19, 20]. When N and calcium (Ca) are deficient in the fruiting zone, their contents in the shell and seed decrease [21, 35]. More than 10% of total N in peanut seeds is absorbed by the pod [24]. However, the behavior of N absorbed through the pod in different peanut cultivars is not well understood. In our study, SH11 showed higher N accumulation than HY23 throughout pod filling and had more N uptake compared to HY23 under the same pod zone N application conditions, except during the first 20 days. N accumulation reached a maximum in SH11 and HY23 at 90 kg·hm^− 2^ and 60 kg·hm^− 2^ pod zone N application, respectively. This may be attributed to the smaller-seeded peanut cultivar having a shorter pod-filling period than the larger-seeded cultivar, resulting in lower yield [36]. Similar results were found in cotton, where large-seeded cultivars were more sensitive in response to N than smaller-seeded cultivars [37].

Nitrate reductase (NR) is a rate-limiting enzyme in the pathway of N absorption and utilization in plants, accelerating N metabolism activity and protein synthesis [38]. Glutamine synthetase (GS) plays a major role in fixing ammonium to form the amino acid glutamine, while glutamate synthase (GOGAT) catalyzes the conversion of glutamine to glutamate, providing glutamate for ammonium assimilation. The activities of GS/GOGAT directly affect the efficiency of N assimilation and play a key role in N metabolism [39, 40]. The activities of NR, NiR, GS, and GOGAT significantly decrease in N deficiency but rapidly recover with supplemental ammonium nitrate.Moderate N application notably promotes yield and enzymatic activities related to N metabolism throughout the growing period in most crops [41, 42]. Excessive N application has adverse effects on NR, GS, and GOGAT activities, leading to reductions in N use efficiency [43, 44]. The activities of NR, GS, GOGAT in the flag leaf were proposed as candidate indicators for rice N accumulation and yield [43]. The Virginia type (large-seeded) peanut cultivar exhibited higher NR activity and N fixation levels than the Spanish type (small-seeded) [45]. In our current study, the increase in pod NR activity was greater in SH11 than in HY23 under pod zone N application compared to control, reaching a maximum at the 60 kg·hm^− 2^ treatment in both cultivars. Pod GS activity in SH11 showed less increase than in HY23 at S1 and S2 compared to control but increased more at S3. The maximum increase in pod GS activities in both cultivars was found under the highest pod zone N application at S2. This might be attributed to the pod development of larger-seeded peanuts being later than that of smaller-seeded peanuts [36], requiring more N for pod development [25]. These results also suggest that N uptake in the pod is most sensitive at S2. GOGAT activity showed a trend similar to GS activity but exhibited an observable decrease in HY23 at S1 compared to control. Our findings are consistent with a field N trial in peanuts [46], where N application enhanced the activities of GS and GOGAT in the root, leaf, and stem, increasing the protein content in the kernel. Larger-seeded peanuts require more N application to maintain high N metabolism activity.

Plant nitrogen (N) nutrition status is regulated by a series of genes associated with N uptake and utilization, with gene expression levels primarily activated by soil N content [47]. In spinach roots and leaves, the expression levels of *NR* and *NiR* genes decrease during N deprivation and increase after optimal N replenishment, but further decrease with excessive N supplementation [48]. N-efficient rice cultivars exhibit higher transcription levels of *OsNRT *in roots and leaves than N-inefficient ones, accumulating more N under N-deficient conditions [49]. N application significantly up-regulates the expression of *NRT*, *NR*, *NiR*, *GS*, and *GOGAT* in rootstock-grafted watermelon compared to self-grafted, enhancing N uptake and utilization [50]. In the present research, the expressions of *NR*, *NiR*, *GS*, *NADH-GOGAT*, *ABC*, and *NRT2* genes in the pod were minimally affected by pod zone N application at S1. These results align with transcriptome expressions during different pod development stages in peanuts [18]. This lack of impact at S1 may be attributed to the immature pod’s weak absorption capacity, which is not sensitive to N response. Maximum up-regulated expressions of the mentioned genes were observed at S3 in SH11 and at S2 in HY23 compared to the control, under 90 kg·hm^− 2^ and 60 kg·hm^− 2^ pod zone N application at S2 and S3 in the two cultivars, respectively. SH11 exhibited higher expressions with these genes than HY23, suggesting a longer duration of N-efficient pod absorption in large-seeded peanut cultivars compared to small-seeded cultivars. These findings are consistent with our pod transcriptome analysis under pod zone N application, indicating increased absorption activity at S2 [24].

Correlation analysis revealed significant positive correlations between yield and the mentioned enzymatic activities and relative gene expressions in SH11 at S2 and S3, and in HY23 at S3 only. Remarkable positive correlations were observed between N accumulation and the mentioned enzymatic activities and relative gene expressions in SH11 at S2 and S3, and in HY23 at S2 only. This suggests that pod zone N supplementation promotes pod N uptake and yield formation to some extent by activating pod nitrogen metabolism. These results align with previous studies that demonstrated significant positive correlations between yield and transcript levels and activities in *NR*, *NiR*, *GS*, and *GOGAT* in wheat [41] and citrus [44]. Over-expression of *NR* and *GS* genes in transgenic wheat [51] and rice [52] significantly enhanced *NR* and *GS* activities in roots and leaves, promoting root capacity to obtain N, increasing grain protein content, yield, and 1000-grain weight compared to the wild type. SH11 exhibited a closer correlation than HY23 between yield and N accumulation in response to pod zone N application at different pod developmental stages, possibly due to the longer pod development period and higher N requirement in large-seeded peanut cultivars compared to small-seeded cultivars [36, 46].

## Conclusions

In summary, the application of nitrogen in the pod zone increased enzyme activities and gene expressions related to nitrogen metabolism in the pod, enhancing yield and nitrogen accumulation in plants. The peanut varieties, SH11 (Var. *hypogaea* type) and HY23 (Var. *vulgaris* type), exhibited distinct responses to nitrogen application in the pod zone. HY23 demonstrated the highest up-regulation in physical traits in response to pod zone N application at S2 compared to the control. On the other hand, SH11 maintained a close sensitivity to pod zone N application at both S2 and S3.The optimal pod zone N application was the 60 kg·hm^− 2^ treatment, as indicated by the positive effects on yield and nitrogen accumulation, with a more pronounced impact on the large-seeded cultivar than the small-seeded one. This knowledge will be valuable for the high-efficiency nitrogen management of different peanut cultivars.

## Data Availability

Data are contained within the article.
